# Association of Mediterranean diet adherence during pregnancy with maternal and neonatal lipid, glycemic and inflammatory markers: The GESTAFIT project

**DOI:** 10.1111/mcn.13454

**Published:** 2022-11-27

**Authors:** Marta Flor‐Alemany, Pedro Acosta‐Manzano, Jairo H. Migueles, Laura Baena‐García, Pilar Aranda, Virginia A. Aparicio

**Affiliations:** ^1^ Department of Physiology University of Granada Granada Spain; ^2^ Institute of Nutrition and Food Technology University of Granada Granada Spain; ^3^ Sport and Health University Research Institute (iMUDS) Granada Spain; ^4^ Department of Physical Education and Sports, Phsical Activity for Health Promotion, CTS‐1018 Research Group, Faculty of Sport Sciences University of Granada Granada Spain; ^5^ Institute of Human Movement Science, Sport and Health University of Graz Graz Austria; ^6^ Department of Biosciences and Nutrition Karolinska Institutet Huddinge Sweden; ^7^ Department of Physical Education and Sports, PROFITH “Promoting FITness and Health through Physical Activity” Research Group, Faculty of Sport Sciences University of Granada Granada Spain; ^8^ Department of Nursing, Faculty of Health Sciences University of Granada Ceuta Spain; ^9^ Instituto de investigación biosanitaria, ibs Granada Granada Spain

**Keywords:** Cholesterol, cord‐blood, Diet, Gestation, HDL, HOMA‐IR, LDL, Newborn

## Abstract

To examine the association of Mediterranean diet (MD) adherence during pregnancy with maternal and neonatal lipid, glycemic, and inflammatory markers. This study included 152 women from the GESTAFIT trial and a subsample of 35 newborns. The Mediterranean Diet Score, derived from food frequency questionnaires, was employed to assess MD adherence. Total cholesterol, high‐density lipoprotein‐cholesterol (HDL‐C), low‐density lipoprotein‐cholesterol (LDL‐C), triglycerides, and glucose were assessed in the mother (at the 16th and 34th gestational weeks [g.w.]) and in cord arterial and venous serum with standard procedures using an autoanalyzer. Pro‐inflammatory and anti‐inflammatory cytokines (interleukin [IL]−6, IL‐8, IL‐10, IL‐1beta, interferon gamma, and tumour necrosis factor alpha [TNF‐α]) were measured with Luminex xMAP technology. A greater MD adherence was associated with higher HDL‐C and lower LDL‐C, LDL‐C/HDL‐C ratio, triglycerides, triglycerides/HDL‐C ratio, and TNF‐α in the mother at the 16th and the 34th g.w. (|β|: 0.191–0.388, *p* < 0.05). A higher intake of whole grain cereals, fruits, vegetables and fish and a lower intake of sweets were associated with higher HDL‐C and lower LDL‐C, LDL‐C/HDL‐C ratio, triglycerides, triglycerides/HDL‐C ratio, and TNF‐α at the 16th and 34th g.w. (|β|: 0.188–0.334, *p* < 0.05). No associations were found with the cord arterial and venous serum markers (*p* > 0.05). A greater MD adherence during pregnancy, driven by a higher intake of whole grain cereals, fruits, vegetables and fish, and a lower intake of sweets, was positively associated with the maternal lipid and inflammatory serum markers throughout gestation. MD adherence during pregnancy was not associated with cord serum markers.

## Key messages

- A greater Mediterranean adherence during pregnancy, driven by a higher intake of fish, whole grain cereals, fruits and vegetables and a lower intake of sweets, was associated with healthier maternal lipid serum markers throughout the pregnancy course.
- Mediterranean adherence during pregnancy was not associated with the maternal glycemic profile.
- Mediterranean adherence during pregnancy was not associated with cord serum markers.

## 1 INTRODUCTION

During pregnancy, essential immunometabolic adaptations occur to sustain pregnancy and promote foetal growth and development (Kalagiri et al., 2016; Yockey & Iwasaki, 2018). Nonetheless, an exacerbation or dysregulation of inflammatory and cardiometabolic markers might lead to a higher risk of developing pregnancy‐related complications such as gestational hypertension, pre‐eclampsia, gestational diabetes, preterm labour, and spontaneous abortion (Kalagiri et al., 2016; Martin et al., 2016; Salazar Garcia et al., 2018; Sheiner et al., 2019; Stokkeland et al., 2019). These complications have adverse outcomes for both the mother and infant, which may appear during pregnancy or at birth, or even suppose a lifelong risk for cardiovascular disease, type‐2 diabetes, and other chronic metabolic conditions for the offspring (Kalagiri et al., 2016).

Maternal dietary intake is one potentially modifiable behaviour that might positively impact materno‐foetal immunometabolic markers (Martin et al., 2016). However, evidence of the whole diet that considers complex nutrient and food interaction when examining materno‐foetal cardiometabolic health is rare and conflicting (Chen et al., 2021; Khoury et al., 2005, 2007). In this context, the Mediterranean diet (MD) might be beneficial for materno‐foetal health (Biagi et al., 2019). For the mother, achieving a healthier dietary pattern, such as the MD, seems to lower the risk for gestational diabetes (Izadi et al., 2016; Mahjoub et al., 2021), gestational hypertension (Schoenaker et al., 2015) and is associated with lower triglycerides (Martin et al., 2016) and fasting blood glucose (Mahjoub et al., 2021) concentrations. As for the foetus, greater maternal MD adherence has been associated with a reduced occurrence of preterm birth, better lipoprotein concentration, and insulin sensitivity measured in the umbilical cord (Biagi et al., 2019; Gesteiro et al., 2012, 2015). To our knowledge, only one previous trial (the CARRDIP, Cardiovascular Risk Reduction Diet in Pregnancy) has investigated the effect of a cholesterol‐lowering diet based on fish, low‐fat meats and dairy products, oils, whole grains, fruits and legumes during pregnancy on materno‐foetal lipid and inflammatory markers (Khoury et al., 2005, 2007). They found that this diet lowered total cholesterol and LDL‐C concentrations in the mothers throughout the pregnancy (Khoury et al., 2005), yet this did not lead to improvements in the maternal or foetal lipid and inflammatory profiles (Khoury et al., 2007).

Further studies considering not only dietary habits but also diet quality during pregnancy, and their relation with both maternal and foetal immunometabolic serum markers, are required to provide more robust evidence on this relevant topic. Therefore, the aim of the present study was to analyze the associations of MD adherence during pregnancy and MD components, with maternal and cord arterial and venous glycemic, lipid and inflammatory serum markers.

## 2 METHODS

### 2.1 Study design and participants

These are secondary analyses of the GEStation and FITness (GESTAFIT) project where a concurrent exercise programme (60 min/session, 3 days/week of combined aerobic and strength training) from the 17th g.w. until delivery of concurrent exercise intervention (i.e., aerobic plus strength training) was conducted (Aparicio et al., 2016). The participants were recruited between the 11th and 13th gestational weeks (g.w.) at the ‘San Cecilio’ University Hospital (Granada, Spain) during their first gynaecologist checkup. The study was carried out at the ‘Sport and Health University Research Institute’ (Granada, Spain) and at the ‘San Cecilio and Virgen de las Nieves University Hospitals’ from November 2015 to April 2018. This study was approved by the Clinical Research Ethics Committee of Granada, Government of Andalusia, Spain (code: GESFIT‐0448‐N‐15). The general assessment procedures are detailed in Supporting Information: Figure S1. Of 384 pregnant women assessed for eligibility, 159 women who met the inclusion–exclusion criteria and accepted to participate (Supporting Information: Table S1) were recruited for the GESTAFIT project. All participants provided a written informed consent. Among them, a total of 152 had valid data in sociodemographic characteristics and MD adherence at the 16th g.w. and were included in the present analyses (Supporting Information: Figure S2). Funding limitations of the project made it unfeasible to collect cord serum samples from all the women included, and thus, a total of 43 women provided cord serum samples for the glycemic, lipid and inflammatory marker assessment. Among them, a total of 35 women had valid data in food frequency questionnaires and, therefore, MD adherence and were included in the present analyses.

#### 2.1.1 Sample size calculation

The sample size for this study depended on the ‘a priori’ analyses of the statistical power performed in the GESTAFIT project (Aparicio et al., 2016). Based on the primary outcome (i.e., maternal weight gains and maternal/neonatal glycemic profiles), we planned to recruit 60 women assuming a statistical power of 90%, *α* = 0.05, and 15% of potential withdrawals. Given the exploratory basis of the present study (secondary outcomes), we did not calculate the sample size.

### 2.2 Outcomes

#### 2.2.1 Maternal anthropometry and body composition

Mothers reported their pre‐pregnancy weight at the recruitment (i.e., 12th g.w.). Although measured weight is preferable, self‐report is a cost‐effective and practical measurement approach that shows very good concordance with measured body weight (Headen et al., 2017). Weight and height were measured at the first and the second contact with the project team (16th g.w. and 34th g.w., respectively). Body weight and height were assessed using a scale (InBody R20; Biospace) and a stadiometer (Seca 22), respectively. Height and pre‐pregnancy weight were used to calculate pre‐pregnancy body mass index, as weight (kg) divided by squared height (m^2^).

#### 2.2.2 Blood collection

Maternal blood samples (5 ml) were extracted from the antecubital vein in standardized fasting conditions (at the 16th and 34th g.w.), and arterial and vein cord blood samples were extracted immediately after delivery. All blood samples were collected in serum tubes and were allowed to clot (coagulation) at room temperature for 30 min. Subsequently, samples were centrifuged at 1750 rpm for 10 min at 4°C in a refrigerated centrifuge (GS−6R Beckman Coulter) to obtain serum, which was aliquoted and frozen at −80°C until analyzed.

#### 2.2.3 Biochemical markers

##### Glycemic profile, lipids and C‐reactive proteins

Maternal concentrations of serum total cholesterol, HDL‐cholesterol, LDL‐cholesterol, glucose, phospholipids, triglycerides and C‐reactive proteins were measured with standard spectrophotometric enzyme assays (AU5822 Clinical Chemistry Analyser, Beckman Coulter). Cord arterial and venous serum total cholesterol, HDL‐cholesterol, LDL‐cholesterol, glucose, triglycerides, and CRP were measured with spectrophotometric determination (BS‐200 Chemistry Analyzer, Mindray Bio‐medical Electronics). In addition, triglycerides/HDL‐cholesterol and LDL‐c/HDL‐c ratios were calculated (Eshriqui et al., 2020; Song et al., 2021).

##### Insulin

Maternal insulin (at the 16th and 34th g.w.) was measured with paramagnetic‐particle‐based chemiluminescence immunoassays (UniCel‐Dxl800 Access Immunoassay analyser, Beckman Coulter).

##### Inflammatory markers

Some maternal and cord arterial and venous pro‐inflammatory and anti‐inflammatory cytokines (IL‐6, IL‐8, IL‐10, IL‐1beta, interferon gamma and TNF‐α*)* were assessed with Luminex xMAP technology. The plate was read on a LABScan 100 analyzer (Luminex Corporation) with xPONENT software for data acquisition. Average values for each set of duplicate samples or standards were within 15% of the mean.

##### Maternal insulin resistance

To assess maternal insulin resistance the homoeostasis model assessment (HOMA)‐IR was employed as previously described (HOMA‐IR = insulin × glucose/22.5) (Matthews et al., 1985).

##### Dietary assessment and Mediterranean diet adherence

Dietary habits were assessed at the 16th and 34th g.w. with a food frequency questionnaire previously evaluated for validity in the Spanish nonpregnant adult population (Mataix et al., 2000). By means of the data obtained from the food frequency questionnaire, the Mediterranean Diet Score (Panagiotakos et al., 2006) was employed to assess MD adherence as previously done in this study sample (Flor‐Alemany et al., 2021). The Mediterranean Diet Score (Panagiotakos et al., 2006) consists of 11 variables (i.e., whole grain cereals, potatoes, fruits, vegetables, pulses, fish, olive oil, red wine, red meat and subproducts, poultry and whole dairy products) ranging from 0 to 5 according to their position in the MD pyramid (Bach‐Faig et al., 2011). Therefore, the total score ranges from 0 to 55. A moderate alcohol intake, also typical of the MD, was not considered for calculating the index in this group of women, since they must not drink alcohol during this period. As a result, the maximum score considered for these analyses in pregnant women was 50 points. Higher scores indicate higher MD adherence and, therefore, higher diet quality. The dietary pattern registered at the 16th g.w. was taken as representative of the pregnancy diet habits as previously done in this study sample (Flor‐Alemany et al., 2022).

### 2.3 Statistical analysis

Descriptive characteristics are presented as means and standard deviations for continuous variables and as frequency and percentages for categorical variables.

As initially designed (Aparicio et al., 2016), statistical analysis was conducted on a per‐protocol basis. We only included women who attended more than 75% of exercise sessions and had valid data in both baseline and follow‐up assessments. The changes (post‐test [34th g.w.]‐pre‐test [16th g.w.]) of MD adherence and MD components were calculated. Changes (post‐test–pre‐test) in dietary variables were included as dependent variables, whereas the exercise intervention (control or exercise group) as independent variables.

Hierarchical linear regression analyses were performed to examine the associations of MD adherence (i.e., explanatory variable) with maternal and neonatal lipid, glycemic and inflammatory serum markers (i.e., glucose, HOMA‐IR, total cholesterol, LDL‐C, HDL‐C, triglycerides, phospholipids, IL‐6, IL‐8, IL‐10, IFN‐γ, TNF‐α and CRP). The stepwise method was used, entering potential confounders (i.e., age, gestational weight gain, and smoking habit) in Step 1 to test their association with each outcome (lipid, glycemic, and inflammatory serum markers in separate models). In Step 2 of the hierarchical regressions, we entered the MD adherence as a predictor and each lipid, glycemic, and inflammatory serum marker as outcomes in separate regression analyses after the inclusion of the relevant confounders (i.e., those that explained a significant amount of the variance in lipid, glycemic and inflammatory serum markers) found in Step 1. These steps were the same in the cross‐sectional association models (i.e., maternal outcomes measured at the 16th g.w.) and in the longitudinal association models (i.e., maternal outcomes measured at the 34th g.w. and neonatal outcomes). The longitudinal models were additionally adjusted for the exercise intervention in Step 2 to account for the possible effect of the intervention conducted within the GESTAFIT project on these outcomes. As an exploratory analysis, additional linear regressions were performed using the MD components as explanatory variables and maternal (at the 16th and 34th g.w.) and neonatal serum markers after adjusting for the confounders previously found relevant in the hierarchal linear regression.

**Table 1:** Anthropometric and clinical characteristics of the study sample

Variable	*N*	Mean (SD)
Age (years)	152	32.9 (4.6)
Pre‐pregnancy weight (kg)	140	65.0 (12.3)
Weight at the 16th g.w. (kg)	150	66.9 (11.9)
Weight at the 34th g.w. (kg)	121	74.6 (10.9)
Gestational weight gain (pre‐pregnancy to 16th g.w.) (kg)	138	2.1 (2.8)
Gestational weight gain (pre‐pregnancy to 34th g.w.) (kg)	116	10.6 (5.0)
Pre‐pregnancy body mass index (kg/m^2^)	138	24.2 (4.2)
Mediterranean diet score (0–50)	152	28.9 (3.9)
Number of children	152	*n* (%)
0		91 (59.9)
1		53 (34.9)
2		8 (5.3)
Educational status	152	
University studies		90 (59.2)
No University studies		62 (40.8)
Marital status	152	
Married		90 (59.2)
Single/divorced/widow		62 (40.8)
Working status	152	
Working		104 (68.4)
Not working		48 (31.6)
Sex of the baby	137	
Male		66 (48.2)
Female		71 (51.8)
Type of delivery	135	
Spontaneous		79 (58.5)
Instrumental vacuum/forceps		22 (16.3)
Caesarean		34 (25.2)
Smoking habit	152	
Never smoker		81 (53.3)
Current smoker		13 (8.6)
Former smoker		58 (38.2)

*Note*: Values shown as mean (standard deviation) unless otherwise indicated.

Abbreviation: g.w., gestational week.

**Table 2:** Cross‐sectional associations of the Mediterranean diet adherence with maternal glycemic, lipid and inflammatory serum markers at the 16th gestational week

Cross‐sectional associations				
	Standardized coefficients (*β*)	Confidence interval (95%)		*p* Value
		Lower	Upper	
Lipid and glycemic profiles				
Glucose (mg/dl) (*n* = 120)^a^	−0.023	−0.250	0.803	0.803
HOMA‐IR (*n* = 117)^a^	0.056	−0.129	0.240	0.550
Total cholesterol (mg/dl) (*n* = 121)^b^	−0.171	−0.347	0.006	0.058
HDL‐cholesterol (mg/dl) (*n* = 121)^c^	0.289	0.126	0.453	**0.001**
LDL‐cholesterol (mg/dl) (*n* = 121)^a^	−0.264	−0.439	−0.089	**0.003**
LDL‐cholesterol/HDL‐cholesterol (*n* = 121)^a^	−0.388	−0.555	−0.221	**<0.001**
Triglycerides (mg/dl) (*n* = 121)^a^	−0.142	−0.321	0.038	0.121
Triglyceride/HDL‐cholesterol (*n* = 121)^a^	−0.235	−0.412	−0.059	**0.009**
Inflammatory profile				
Interleukin 6 (pg/ml) (*n* = 46)^a^	−0.069	−0.372	0.234	0.647
Interleukin 8 (pg/ml) (*n* = 46)^a^	0.261	−0.032	0.554	0.080
Interleukin 10 (pg/ml) (*n* = 46)^a^	0.214	−0.083	0.510	0.154
Interleukin 1 beta (pg/ml) (*n* = 46)^a^	0.038	−0.266	0.341	0.803
Interferon gamma (pg/ml) (*n* = 46)^a^	0.206	−0.091	0.504	0.178
Tumour necrosis factor alpha (pg/ml) (*n* = 46)^a^	0.117	−0.185	0.418	0.690
C‐reactive protein (*n* = 120)^a^	−0.028	−0.210	0.761	0.761

Abbreviations: HDL‐C, high‐density lipoprotein cholesterol; LDL‐C, low‐density lipoprotein cholesterol.

^a^ Unadjusted.

^b^ Adjusted by age.

^c^ Adjusted by age, gestational weight gain (body weight at the 16th gestational week‐pre‐pregnancy body weight), and smoking habit. Boldface indicates those outcomes that surpassed the multiple comparison test.

**Table 3:** Longitudinal associations of the Mediterranean Diet Score with maternal glycemic, lipid and inflammatory serum markers at the 34th gestational week

Prospective associations				
	Standardized coefficients (β)	Confidence interval (95%)		*p* Value
		Upper	Upper	
Lipid and glycemic profiles				
Glucose (mg/dl) (*n* = 108)^a^	−0.164	−0.356	0.028	0.093
HOMA‐IR (*n* = 107)^b^	−0.097	−0.287	0.092	0.312
Total cholesterol (mg/dl) (*n* = 108)^a^	0.080	−0.114	0.273	0.414
HDL‐cholesterol (mg/dl) (*n* = 108)^c^	0.348	0.173	0.522	**<0.001**
LDL‐cholesterol (mg/dl) (*n* = 108)^a^	0.065	‐0.129	0.258	0.509
LDL‐cholesterol/HDL‐cholesterol (*n* = 108)^a^	−0.191	−0.381	−0.001	0.049
Triglycerides (mg/dl) (*n* = 108)^a^	−0.195	−0.384	‐0.005	0.044
Triglyceride/HDL‐cholesterol (*n* = 108)^a^	−0.293	−0.476	−0.109	**0.002**
Inflammatory profile				
Interleukin 6 (pg/ml) (*n* = 49)^a^	−0.113	−0.408	0.181	0.443
Interleukin 8 (pg/ml) (*n* = 49)^a^	−0.129	−0.423	0.165	0.382
Interleukin 10 (pg/ml) (*n* = 49)^c^	−0.190	−0.459	0.079	0.162
Interleukin 1 beta (pg/ml) (*n* = 49)^a^	−0.047	−0.340	0.245	0.747
Interferon gamma (pg/ml) (*n* = 49)^a^	0.121	−0.172	0.414	0.411
Tumour necrosis factor alpha (pg/ml) (*n* = 49)^a^	−0.299	−0.573	−0.024	0.034
C‐reactive protein (*n* = 107)^a^	−0.020	−0.215	0.174	0.836

Abbreviations: HDL‐C, high‐density lipoprotein cholesterol; LDL‐C, low‐density lipoprotein cholesterol.

^a^ Adjusted by exercise intervention.

^b^ Adjusted by exercise intervention and gestational weight gain (weight at the 34th gestational week‐pre‐pregnancy body weight).

^c^ Adjusted by exercise intervention and age. Boldface indicates those outcomes that surpassed the multiple comparison test.

The Benjamini–Hochberg procedure (Benjamini & Hochberg, 1995) was applied to account for the random effect in multiple comparisons for all the tests included in the primary analysis (i.e., MD adherence associations with materno‐foetal immunometabolic serum markers at the 16th and 34th g.w.) and separately for all the tests included in the MD component analysis (i.e., MD component associations with maternal immunometabolic serum markers at the 16th and 34th g.w.) with *q* = 0.05 (false discovery rate).

All analyses were conducted using the Statistical Package for Social Sciences (IBM SPSS Statistics for Windows, version 22.0), and the level of significance was set at *p* ≤ 0.05.

### 2.4 Ethical statement

The GESTAFIT project was approved by the Clinical Research Ethics Committee of Granada, Government of Andalusia, Spain (code: GESTAFIT‐0448‐N‐15, approved on 19/05/2015).

## 3 RESULTS

The total sample size for the present analyses comprised 152 Caucasian pregnant women (32.9 ± 4.7 years, pre‐pregnancy body mass index 24.2 ± 4.2 kg/m^2^) who had valid data in sociodemographic characteristics and MD adherence at the 16th g.w. (Supporting Information: Figure S2). The sociodemographic characteristics of the study sample are shown in Table 1.

**Table 4:** Linear regression analysis assessing the association of maternal Mediterranean diet adherence with cord arterial and venous serum markers

Artery umbilical cord blood	Standardized coefficients (*β*)	Confidence interval (95%)		*p* Value
		Upper	Upper	
Umbilical arterial serum				
Lipid and glycemic profiles				
Glucose (mg/dl) (*n* = 24)^a^	0.094	−0.296	0.485	0.619
Total cholesterol (mg/dl) (*n* = 24)^b^	−0.076	−0.483	0.331	0.702
HDL‐cholesterol (mg/dl) (*n* = 24)^b^	−0.363	−0.768	0.042	0.077
LDL‐cholesterol (mg/dl) (*n* = 24)^b^	0.096	−0.318	0.510	0.634
LDL‐cholesterol/HDL‐cholesterol (*n* = 24)^b^	0.311	−0.118	0.740	0.146
Triglycerides (mg/dl) (*n* = 22)^c^	0.243	−0.178	0.663	0.241
Triglyceride/HDL‐cholesterol (*n* = 22)^d^	0.211	−0.185	0.607	0.276
Inflammatory profile				
Interleukin 6 (pg/ml) (*n* = 31)^e^	0.009	−0.318	0.335	0.956
Interleukin 8 (pg/ml) (*n* = 31)^e^	0.188	−0.206	0.582	0.338
Interleukin 10 (pg/ml) (*n* = 31)^e^	0.207	−0.168	0.583	0.267
Interleukin 1 beta (pg/ml) (*n* = 31)^e^	−0.101	−0.496	0.293	0.603
Interferon gamma (pg/ml) (*n* = 31)^e^	0.077	−0.302	0.456	0.680
Tumour necrosis factor alpha (pg/ml) (*n* = 31)^e^	0.032	−0.350	0.413	0.866
Umbilical venous serum				
Lipid and glycemic profiles				
Glucose (mg/dl) (*n* = 32)^e^	−0.075	−0.438	0.289	0.677
Total cholesterol (mg/dl) (*n* = 32)^e^	0.107	−0.273	0.486	0.570
HDL‐cholesterol (mg/dl) (*n* = 32)^f^	0.094	−0.253	0.440	0.584
LDL‐cholesterol (mg/dl) (*n* = 32)^e^	0.043	−0.339	0.424	0.821
LDL‐cholesterol/HDL‐cholesterol^e^	−0.052	−0.411	0.307	0.769
Triglycerides (mg/dl) (*n* = 29)^d^	0.065	−0.294	0.424	0.712
Triglyceride/HDL‐cholesterol (*n* = 29)^d^	0.096	−0.287	0.478	0.611
Inflammatory profile				
Interleukin 6 (pg/ml) (*n* = 35)^a^	0.011	−0.344	0.366	0.949
Interleukin 8 (pg/ml) (*n* = 35)^e^	−0.021	−0.388	0.345	0.906
Interleukin 10 (pg/ml) (*n* = 35)^g^	0.147	−0.196	0.489	0.389
Interleukin 1 beta (pg/ml) (*n* = 35)^e^	0.307	−0.044	0.657	0.084
Interferon gamma (pg/ml) (*n* = 35)^c^	0.143	−0.194	0.481	0.393
Tumour necrosis factor alpha (pg/ml) (*n* = 35)^e^	0.234	−0.099	0.568	0.162

Abbreviations: HDL‐C, high‐density lipoprotein cholesterol; LDL‐C, low‐density lipoprotein cholesterol.

^a^ Adjusted by exercise intervention and age.

^b^ Adjusted by exercise intervention and sex of the baby.

^c^ Adjusted by exercise intervention and type of delivery.

^d^ Adjusted by exercise intervention, age and type of delivery.

^e^ Adjusted by exercise intervention.

^f^ Adjusted by exercise intervention and gestational weight gain.

^g^ Adjusted by exercise intervention and smoking habit.

Differences in MD adherence and MD components between pre‐ and post‐intervention for the control and exercise groups (per protocol basis) are shown in Supporting Information: Table S2. No changes were observed regarding MD adherence between the control and exercise groups. Hierarchical linear regression analyses for the association of MD adherence with maternal glycemic, lipid, and inflammatory markers at the 16th g.w. are presented in Table 2. A greater MD adherence was associated with higher HDL‐C (*β* = 0.289, *p* = 0.001), lower LDL‐C (*β* = −0.264, *p* = 0.003), LDL‐C/HDL‐C ratio (*β* = −0.388, *p* < 0.001), and lower triglyceride/HDL‐C ratio (*β* = −0.235, *p* = 0.009). There was evidence of statistical significance in the association of MD adherence with the total cholesterol level (*β* = −0.171, *p* = 0.058). After correcting for multiplicity, we observed that all the cross‐sectional associations between MD adherence and maternal immunometabolic serum markers remained significant.

Hierarchical linear regression analyses for the association of MD adherence with maternal glycemic, lipid, and inflammatory markers at the 34th g.w. are presented in Table 3. A greater MD adherence was associated with higher HDL‐C (*β* = 0.348, *p* < 0.001), lower LDL‐C/HDL‐C ratio (*β* = −0.191, *p* = 0.049), triglycerides (*β* = −0.195, *p* = 0.044), triglyceride/HDL‐C ratio (*β* = −0.293, *p* = 0.002), and lower TNF‐α (*β* = −0.299, *p* = 0.034). After the multiple test correction, the associations between MD adherence, HDL‐C and triglyceride/HDL‐C ratio remained significant.

No associations were found between MD adherence and cord arterial and venous serum glycemic, lipid and inflammatory markers (Table 4) (all, *p* > 0.05).

Table 5 shows the associations between the MD components and the maternal serum markers that were associated with the MD adherence at the 16th and 34th g.w. At the 16th g.w., a greater intake of whole grain cereals, fruits, and fish and a lower intake of sweets were associated with greater HDL‐C, lower LDL‐C, LDL‐C/HDL‐C ratio and lower triglycerides/HDL‐C ratio (|β|ranging from 0.188 to 0.281, all *p* < 0.05). At the 34th g.w., a greater intake of whole grain cereals, vegetables and fish was associated with greater HDL‐C, lower LDL‐C/HDL‐C ratio, triglycerides, lower triglycerides/HDL‐C ratio and lower TNF‐α (|β|ranging from 0.193 to 0.334, all *p* < 0.05). After correcting for multiplicity, we observed that the cross‐sectional association between fish, HDL‐C, and LDL‐C/HDL‐C ratio remained significant. Additionally, the longitudinal associations between vegetables, fish and HDL‐C and the association between fish and triglycerides‐HDL‐C ratio remained significant.

**Table 5:** Association of Mediterranean diet components with maternal glycemic, lipid and inflammatory serum markers at the 16th and 34th gestational weeks

Food groups (servings/week)	Cross‐sectional associations								Longitudinal associations									
	HDL‐cholesterol (*n* = 121)		LDL‐cholesterol (*n* = 121)		LDL‐cholesterol /HDL‐cholesterol (*n* = 121)		Triglycerides/HDL‐ cholesterol (*n* = 121)		HDL‐ cholesterol (*n* = 108)		LDL‐cholesterol /HDL‐cholesterol (*n* = 108)		Triglycerides (*n* = 108)		Triglycerides/HDL‐ cholesterol (*n* = 108)		Tumour necrosis factor alpha (*n* = 49)	
	*β*	*p* Value	*β*	*p* Value	*β*	*p* Value	*β*	*p* Value	*β*	*p* Value	*β*	*p* Value	*β*	*p* Value	*β*	*p* Value	*β*	*p* Value
Whole grain cereals	0.015	0.862	−0.092	0.315	−0.051	0.576	−0.188	0.039	0.029	0.761	0.019	0.845	−0.180	0.064	‐0.138	0.156	−0.292	0.038
White bread and rice	0.008	0.928	−0.031	0.736	−0.029	0.749	0.172	0.060	−0.051	0.591	−0.083	0.393	0.092	0.341	0.075	0.439	0.247	0.083
Potatoes	−0.062	0.483	−0.127	0.166	−0.070	0.446	0.013	0.886	0.105	0.271	−0.101	0.301	−0.060	0.539	−0.075	0.437	−0.202	0.156
Fruits	0.139	0.113	−0.136	0.137	−0.215	0.018	−0.122	0.181	0.139	0.145	−0.129	0.185	−0.031	0.752	−0.121	0.208	−0.188	0.191
Vegetables	0.039	0.654	−0.025	0.782	−0.046	0.617	0.011	0.903	0.263	**0.004**	−0.154	0.112	−0.127	0.190	−0.230	0.016	−0.334	0.017
Pulses	0.049	0.576	−0.129	0.160	−0.141	0.123	0.057	0.534	0.154	0.101	−0.188	0.052	−0.031	0.753	−0.084	0.385	−0.062	0.665
Fish	0.272	**0.002**	−0.107	0.242	−**0.281**	**0.002**	−0.202	0.027	0.305	**0.001**	−0.193	0.050	−0.156	0.111	−0.277	**0.004**	−0.239	0.117
Red meat and sob.	−0.068	0.443	0.173	0.058	0.159	0.082	0.085	0.355	0.038	0.686	0.101	0.303	0.025	0.795	0.005	0.955	0.023	0.874
Poultry	−0.060	0.490	0.013	0.890	0.040	0.666	−0.008	0.926	0.029	0.760	−0.063	0.519	−0.077	0.429	−0.069	0.478	−0.212	0.147
Dairy products	0.024	0.783	−0.089	0.334	−0.084	0.357	0.011	0.901	−0.020	0.832	−0.119	0.223	−0.009	0.930	−0.009	0.925	0.062	0.670
Olive oil	0.019	0.836	−0.017	0.851	−0.010	0.914	0.164	0.072	0.037	0.696	−0.149	0.124	0.166	0.086	0.114	0.239	−0.106	0.466
Nuts	0.144	0.096	−0.055	0.547	−0.120	0.190	−0.029	0.754	0.104	0.282	−0.125	0.213	0.131	0.187	0.042	0.676	−0.092	0.533
Sweets	0.078	0.385	0.216	0.017	0.135	0.140	0.031	0.736	−0.046	0.634	0.099	0.312	0.128	0.187	0.162	0.091	−0.039	0.788

*Note*: Model adjusted for the cofounders previously found significantly associated with the outcomes in the hierarchical linear regression. Boldface indicates those outcomes that surpassed the multiple comparison test.

Abbreviations: HDL‐C, high‐density lipoprotein cholesterol; LDL‐C, low‐density lipoprotein cholesterol; Sb, subproducts.

## 4 DISCUSSION

Our results suggest that a greater MD adherence during pregnancy, which seems to be driven by a higher intake of fish, whole grain cereals, fruits and vegetables and a lower intake of sweets, was associated with better maternal lipid serum markers (i.e., lower total cholesterol, higher HDL‐C and lower LDL‐C, LDL‐C/HDL‐C ratio and triglyceride/HDL‐C ratio) throughout the gestation. Notwithstanding, a greater MD adherence during pregnancy was not associated with cord arterial and venous serum markers.

Our findings of an association between MD adherence and maternal cardiometabolic markers throughout gestation were generally consistent with results from other studies (Asemi et al., 2013; Khoury et al., 2005; Martin et al., 2016). Previous randomized controlled trials (Asemi et al., 2013; Khoury et al., 2005; Killeen et al., 2021) and observational studies (Eshriqui et al., 2020; Martin et al., 2016) found an association between healthier maternal dietary patterns and lower total cholesterol, higher HDL‐C, lower LDL‐C, LDL‐C/HDL‐C ratio and triglycerides. Regarding MD adherence, pregnant women in the second trimester of pregnancy (22–26th g.w.) who had a high MD adherence showed lower triglycerides, LDL and total cholesterol compared with pregnant women with a low MD adherence (Šarac et al., 2021). In line with previous studies that showed the beneficial effect of MD adherence regarding blood lipid control (Neuenschwander et al., 2019; Tuttolomondo et al., 2019), we verified that a greater MD adherence during gestation was associated with lower LDL‐C concentrations, higher HDL‐C, as well as lower LDL‐C/HDL‐C ratio, triglycerides, cholesterol, and triglyceride/HDL‐C ratio values at the 16th and 34th g.w., thus indicating a favourable lipid profile. Moreover, we examined which MD components explained the associations between MD adherence and maternal lipid serum markers. We found that a greater intake of fish (mainly driven by white fish [1.3 servings/week], blue fish [1.0 servings/week], canned fish [1.0 servings/week], and shellfish [0.6 servings/week]), whole grain cereals, fruits, and vegetables and a lower intake of sweets was associated with higher HDL‐C, lower LDL‐C, LDL‐C/HDL‐C ratio, triglycerides and triglyceride/HDL‐C ratio. Although our cross‐sectional design does not allow inferring causality, these significant associations in pregnant women, without cardiometabolic diseases, are suggestive of the role of diet in the lipid profile. In agreement, a multiethnic cohort study showed that a ‘healthy’ dietary pattern, characterized by high intakes of whole grains, fruit, dairy, vegetables and unsaturated cooking oil, was inversely associated with total cholesterol, LDL‐C and triglycerides concentrations and positively associated with HDL‐C concentration (Whitton et al., 2018). Furthermore, women with a dietary pattern characterized by high intakes of fruits, yogurt, nuts, vegetables, whole grain cereals and legumes, among other components, were inversely associated with LDL‐C, LDL‐c/HDL‐c ratio and triglycerides (Eshriqui et al., 2020). On the contrary, a dietary pattern characterized by high intakes of snacks, processed meat, fast food, alcoholic beverages and sweets was directly associated with LDL‐C in adult women (Eshriqui et al., 2020). The higher intake of fruits, vegetables, nuts and whole grains in these patterns should have contributed to greater dietary fibre and phytochemical contents, known to be beneficial to risk factors such as LDL‐C (Anderson & Hanna, 1999). On the contrary, a combination of unhealthier dietary habits in the ‘Processed’ dietary pattern could explain the deleterious association found (Eshriqui et al., 2020; Gershuni, 2018).

Besides, in the present study, a greater MD adherence was associated with lower concentrations of TNF‐*α*. These results are in line with the conclusion that the MD exerts its anti‐inflammatory and immunomodulatory effect by decreasing pro‐inflammatory interleukins such as TNF‐*α* and its receptors (Calder et al., 2011; Estruch, 2010). Moreover, it was proven that green vegetables and fruits rich in folate, flavonoids, and antioxidants can significantly reduce the concentrations of inflammatory serum markers, such as TNF‐α (Holt et al., 2009). Similarly, we found that a greater intake of whole grain cereals and vegetables was associated with lower concentrations of TNF‐α at the 34th g.w. To the authors' knowledge, this study is the first to report such an association, which might be relevant to attenuate the pregnancy‐induced inflammation processes. However, this finding should be taken with caution since this association was disregarded by the multiple comparison test, and future studies with higher sample sizes should therefore confirm or contrast this finding.

The lack of association between MD adherence and serum glucose and HOMA‐IR in our study agrees with previous studies (Siervo et al., 2015; Starling et al., 2017) but differs from others (Asemi et al., 2013; Martin et al., 2016; Mompeo et al., 2021; Villegas et al., 2004). In this context, a systematic review and meta‐analysis have not shown the effect of the Dietary Approaches to Stop Hypertension (DASH) on fasting blood glucose and insulin resistance in nonpregnant adult population (Siervo et al., 2015). Among pregnant women, adherence to a dietary pattern characterized by an intake of poultry, nuts, cheese, and whole grains was not associated with maternal fasting glucose (Starling et al., 2017). Notwithstanding, other studies in pregnant women (Asemi et al., 2013; Martin et al., 2016) at the 24–29th g.w. showed that the DASH score‐based method and a dietary pattern characterized by high consumption of fruits, vegetables, whole grains, low‐fat dairy, breakfast bars and water have been associated with lower maternal glucose, insulin and HOMA‐IR (Martin et al., 2016). However, different g.w. in the measurements may partially account for the differing findings. Furthermore, there is a lack of uniformity between indices to assess diet quality during pregnancy (Papazian et al., 2019), specifically in the number of components including classification categories for each item; measurement scales; statistical parameters (mean, median or quintiles of daily intake); and the contribution of each component (positive or negative) to the total score (Aoun et al., 2019; Milà‐Villarroel et al., 2011). The DASH diet index is a sample quintile‐based sum score of seven components (fruits, vegetables, dairy, meat, poultry, fish, and eggs; nuts, seeds, and legumes; fats and oils; and sodium) (Yu et al., 2014), whereas the *Mediterranean Diet Score* is based on 11 food groups (nonrefined cereals, fruits, vegetables, potatoes, legumes, olive oil, fish, red meat, poultry, full‐fat dairy products and alcohol) that include predefined portions and absolute cut‐offs for each food group and assesses the frequency of consumption per month (Panagiotakos et al., 2006). This complicates the comparability between these two indices and could explain, at least in part, the heterogeneity of findings in dietary patterns research during pregnancy. We did not observe an association between MD adherence, MD components, and HOMA‐IR throughout pregnancy. Although the mechanisms explaining associations between the maternal dietary patterns, insulin resistance, and triglyceride concentrations are still partially unclear, the results are biologically plausible. According to previous evidence (Martin et al., 2016), healthier dietary patterns (e.g., tertile 3 in the DASH diet) have higher intakes of fruits, vegetables and whole grains. These food groups are rich sources of antioxidants, phytochemicals, vitamin C, and dietary fibre, which may contribute to the protective associations seen in their study. Discrepancies between the association of dietary patterns with maternal cardiometabolic markers might be partially explained by differences in the ethnicity of the sample population given the fact that the DASH dietary pattern and its association with cardiometabolic profiles differed by country, with significant within‐country variations by sociodemographic characteristics (Tiong et al., 2018). However, it is likely due to large methodological differences, such as reverse causation given the cross‐sectional design, the method of assessing diet quality, the choice of covariates and variation in sample sizes and sample characteristics, and resulting statistical power.

Little information is available on the effects of maternal diet during pregnancy on the offspring's glycemic, lipid and inflammatory serum markers with previous studies reporting mixed findings (Gesteiro et al., 2012, 2015; Khoury et al., 2005). Previous studies (Gesteiro et al., 2012, 2015) found that pregnant women with higher MD adherence during the first trimester of pregnancy (12–15th g.w.) had infants with lower glycemia, insulinemia, HOMA‐IR and LDL‐C compared with women with low MD adherence. The well‐documented transference of placental lipids suggests that a maternal diet with a low MD score negatively affects neonatal LDL‐cholesterol (Gesteiro et al., 2015). However, we did not find differences between neonatal LDL‐cholesterol and the degree of adherence to the MD diet during gestation. In agreement with our results, Killeen et al. (2021) found no associations between the maternal inflammatory potential of the diet and triglycerides, HDL‐C, LDL‐C, total cholesterol and glucose in the cord blood. Furthermore, a maternal diet that promoted fish, low‐fat meats and dairy products, oils, whole grains, fruits, vegetables, and legumes decreased maternal cholesterol concentrations, but not cord and neonatal lipids or inflammatory markers (Khoury et al., 2005, 2007), which is in agreement with our findings.

### 4.1 Limitations and strengths

Some limitations need to be highlighted. First, the observational design does not allow a clear cause–effect identification. Second, the results should be interpreted cautiously, as we could be limited to detect small association sizes. Larger studies should further explore these associations to corroborate our results. Third, the participants were enroled in an exercise intervention that might affect our findings. However, we included the group allocation as a confounder in our longitudinal analyses. Furthermore, we observed that the GESTAFIT exercise intervention did not have any effect on the MD adherence, nor on any of its components (except for legumes). Thus, the primary exposure (i.e., exercise) is not likely to have affected our findings. Fourth, the limited data (*n* = 35) in our study regarding cord blood could bias our findings. However, we found no differences in the baseline characteristics between women who provided cord serum samples and those who did not. Additionally, although our findings were corrected for multiple comparison testing, the likelihood of making a type I error might not be completely disregarded and future studies should confirm our findings. Several strengths of this study are worth considering. A detailed definition of the dietary habits and a valid assessment of the MD diet adherence were employed. Furthermore, lipid, glycemic, and inflammatory serum markers were assessed during the second and the third trimester of pregnancy and in the arterial and vein cord blood, which provides a more comprehensive understanding of materno‐foetal immunometabolic serum markers along the pregnancy course.

## 5 CONCLUSION

Our results suggest a positive role of MD adherence during pregnancy on maternal lipid serum markers. Likewise, MD adherence might be positively associated with the inflammatory marker TNF‐alpha, although this finding should be corroborated in future studies. However, a greater MD adherence during pregnancy was not associated with neonatal lipid, glycemic, and inflammatory markers. Future studies are warranted to confirm these associations and determine the underlying mechanisms.

## AUTHOR CONTRIBUTIONS

Marta Flor‐Alemany was involved in conceptualization, formal analysis, methodology, validation, investigation, data curation, writing, original draft preparation, review and editing of the manuscript. Pedro Acosta‐Manzano was involved in the validation, investigation, data curation, writing, review and editing of the manuscript. Jairo H. Migueles was involved in the formal analysis, validation, data curation, writing, review and editing. Laura Baena‐García was involved in the, writing, review and editing of the manuscript. Pilar Aranda was involved in validation, writing, review and editing of the manuscript. Virginia A. Aparicio was involved in conceptualization, methodology, validation, resources, data curation, writing, review and editing, project administration and funding acquisition.

## CONFLICT OF INTEREST

The authors declare no conflict of interest.

## Supporting information

Supporting information.

## Data Availability

The data sets used and/or analyzed during the current study are available from the corresponding author on reasonable request.
