# Erratum to “Association of Mediterranean diet adherence during pregnancy with maternal and neonatal lipid, glycemic and inflammatory markers: The GESTAFIT project”

**DOI:** 10.1111/mcn.13611

**Published:** 2024-01-04

This article was intended for this special issue, Nutrition in a Lifecourse Perspective, but was inadvertently published in an earlier issue of *Maternal & Child Nutrition*, 19:2. https://onlinelibrary.wiley.com/doi/10.1111/mcn.13454. The publisher apologises for this error and for any confusion it may cause.

When citing this article, please cite it as per its original publication in issue 19:2 as shown here.

Flor‐Alemany, M., Acosta‐Manzano, P., Migueles, J. H., Baena‐García, L., Aranda, P., & Aparicio, V. A. (2023). Association of Mediterranean diet adherence during pregnancy with maternal and neonatal lipid, glycemic and inflammatory markers: The GESTAFIT project. *Maternal & Child Nutrition, 19*, e13454. https://doi.org/10.1111/mcn.13454

