# Supplementary material for: Association of Mediterranean diet adherence during pregnancy with maternal and neonatal lipid, glycemic and inflammatory markers: The GESTAFIT project
Source: Matern Child Nutr. 2022 Nov 27;19(2):e13454. doi: 10.1111/mcn.13454 (PMC10019045; doi:10.1111/mcn.13454)
Supplement: Supplementary file 1 — Supporting information. [file MCN-19-e13454-s001.docx]

**Supplementary Material**

Supplement to: Flor-Alemany et al. “**Influence of the Mediterranean Diet adherence during pregnancy on maternal and neonatal lipid, glycemic and inflammatory profiles. The GESTAFIT project.”**

**Table of contents**

**Supplementary Table S1.** Inclusion and exclusion criteria in the GESTAFIT project.

**Supplementary Figure S1.** General assessment procedures.

**Supplementary Figure S2.** Flow diagram of the study participants.

**Supplementary Table S2.** Influence of the exercise intervention on Mediterranean diet adherence and Mediterranean diet components.

**Supplementary Table S1.** Inclusion and exclusion criteria in the GESTAFIT project.

| ***Inclusion criteria*** |
| --- |
| - Pregnant women aged 25-40 years old with a normal pregnancy course. |
| - Answering “no” to all questions on the PARmed-X for pregnancy.  - Being able to walk without assistance.  - Being able to read and write properly.  - Informed consent: Being capable and willing to provide written consent. |
| ***Exclusion criteria*** |
| - Having acute or terminal illness.  - Having malnutrition.  - Being unable to conduct tests for assessing physical fitness or exercise during pregnancy.  - Having pregnancy risk factors (such as hypertension, type 2 diabetes, etc.).  - Having a multiple pregnancies.  - Having chromosopathy or foetal malformations.  - Having uterine growth restriction.  - Having foetal death.  - Having upper or lower extremity fracture in the past 3 months.  - Suffering neuromuscular disease or presence of drugs affecting neuromuscular function.  - Being registered in another exercise program.  - Performing more than 300 minutes of at least moderate physical activity per week.  -Being engaged in another physical exercise program  - Being unwilling either to complete the study requirements or to be randomized into the control or intervention group. |

**Recruitment at Hospital**

- Inclusion and exclusion criteria
- Written informed consent
- Self-reported pre-pregnancy weight

**First assessment**

*First day evaluation:*

- Weight and height status
- Sociodemographic and clinical data
- Nutritional assessment

*Second day evaluation (week 17^th^).*

- Blood sampling

**Start of the intervention program**

**Second assessment**

*First day evaluation:*

- Weight and height status
- Nutritional assessment

*Second day evaluation (week 34^th^):*

- Blood sampling

**Sample collection at Hospital**

- Arterial and venous umbilical cord blood

**Week of gestation**

**Delivery**

**12**

**16**

**33**

**Supplementary Figure S1.** General assessments procedures.


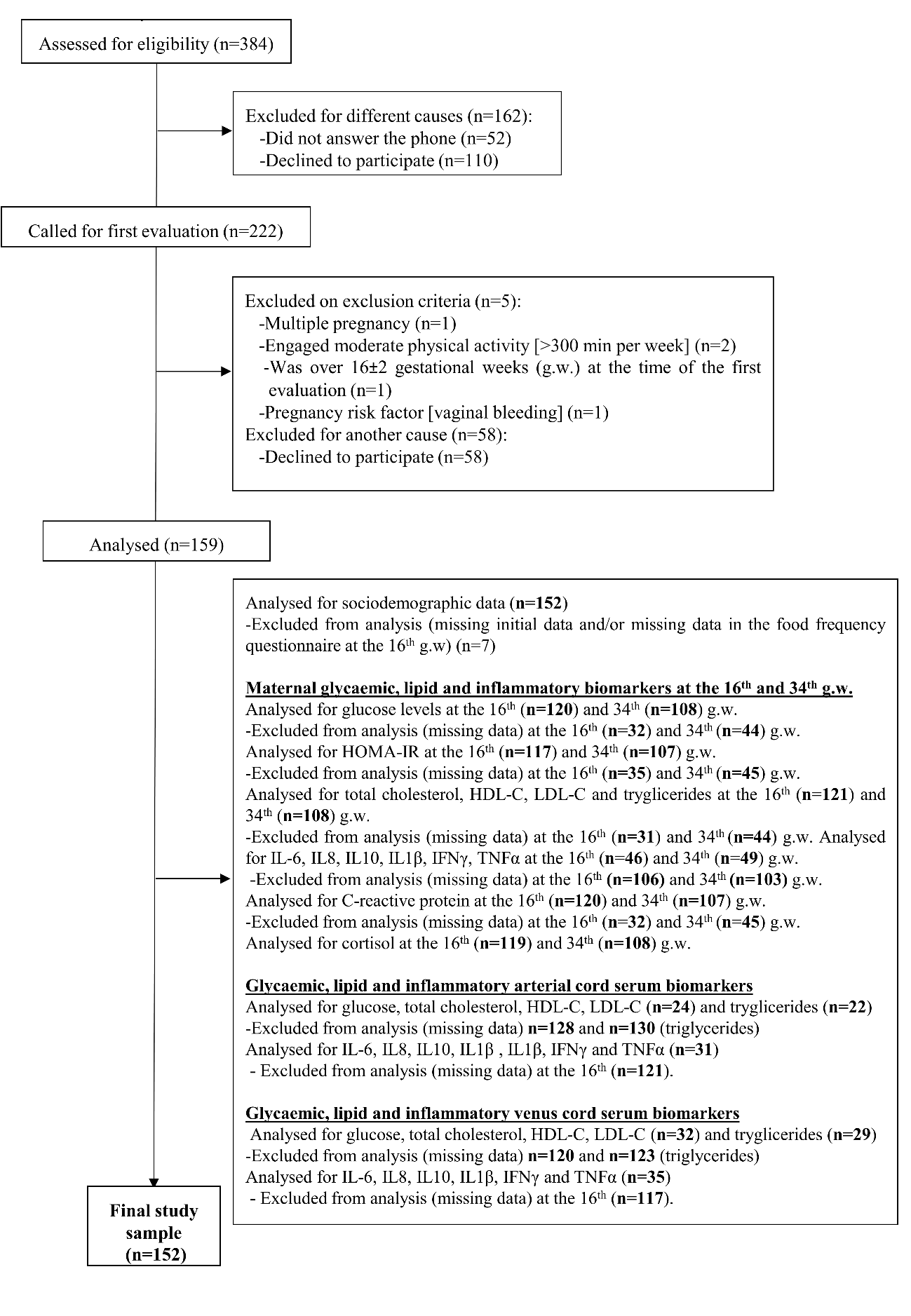


**Supplementary Figure S2**. Flow diagram of study participants.

**Supplementary Table S2.** Influence of the exercise intervention on Mediterranean diet adherence and Mediterranean diet components.

|  | **Within-group changes**  **(Post-Pre)** | |  | **B** | **Confindence Interval (95%) for B** | | **β** | ***P*** |
| --- | --- | --- | --- | --- | --- | --- | --- | --- |
|  | **Control group**  **(n=51)** | **Exercise group (n=46)** |  |  | **Lower** | **Upper** |  |  |
| **Food groups (servings/week)** |  |  |  |  |  |  |  |  |
| *Whole-grain cereals* | 1.9 (3.4) | -0.1 (6.1) |  | -1.521 | -3.541 | 0.499 | -0.154 | 0.138 |
| *White bread and rice* | 1.6 (5.2) | 0.3 (6.6) |  | -1.194 | -3.444 | 1.056 | -0.101 | 0.295 |
| *Potatoes* | -0.1 (1.6) | 0.2 (1.3) |  | 0.174 | -0.377 | 0.724 | 0.059 | 0.533 |
| *Fruits* | 2.9 (6.9) | 0.7 (7.8) |  | -1.057 | -3.769 | 1.654 | -0.072 | 0.441 |
| *Vegetables* | 2.2 (8.4) | 2.1 (7.8) |  | 0.203 | -2.890 | 3.297 | 0.013 | 0.897 |
| *Pulses* | 0.2 (1.0) | -0.2 (0.9) |  | -0.380 | -0.735 | -0.026 | -0.193 | 0.036 |
| *Fish* | 0.2 (2.0) | 0.6 (2.0) |  | 0.719 | -0.024 | 1.462 | 0.178 | 0.058 |
| *Red meat and sb.* | -0.03 (2.76) | 0.2 (2.8) |  | 0.256 | -0.716 | 1.229 | 0.047 | 0.602 |
| *Poultry* | -0.1 (2.0) | -0.03 (2.4) |  | 0.354 | -0.459 | 1.167 | 0.080 | 0.390 |
| *Dairy products* | 1.1 (3.5) | 0.8 (4.1) |  | -0.350 | -1.787 | 1.087 | -0.046 | 0.630 |
| *Olive oil* | 0.8 (10.3) | 0.1 (6.4) |  | -0.397 | -3.553 | 2.759 | -0.023 | 0.803 |
| *Nuts* | 2.6 (4.8) | -0.9 (6.5) |  | -1.812 | -4.031 | 0.407 | -0.153 | 0.108 |
| *Sweets* | 0.5 (5.9) | 0.3 (4.1) |  | -0.208 | -2.250 | 1.834 | -0.020 | 0.840 |
| **Mediterranean Diet Score (0-50)** | 0.5 (2.8) | -0.2 (3.0) |  | -0.437 | -1.532 | 0.658 | -0.074 | 0.430 |

Values shown as mean (standard error). Model adjusted for baseline values (i.e.,16^th^ gestational week). Within-group changes show the differences between post-pre intervention results for each variable with negative values indicating a reduction in the post evaluation compared to pre-evaluation; β. standardized beta. CI, confidence interval; S, serving.
